# Supplementary figures and images for: Efficient neural decoding of self-location with a deep recurrent network
Source: PLoS Comput Biol. 2019 Feb 15;15(2):e1006822. doi: 10.1371/journal.pcbi.1006822 (PMC6407788; doi:10.1371/journal.pcbi.1006822)

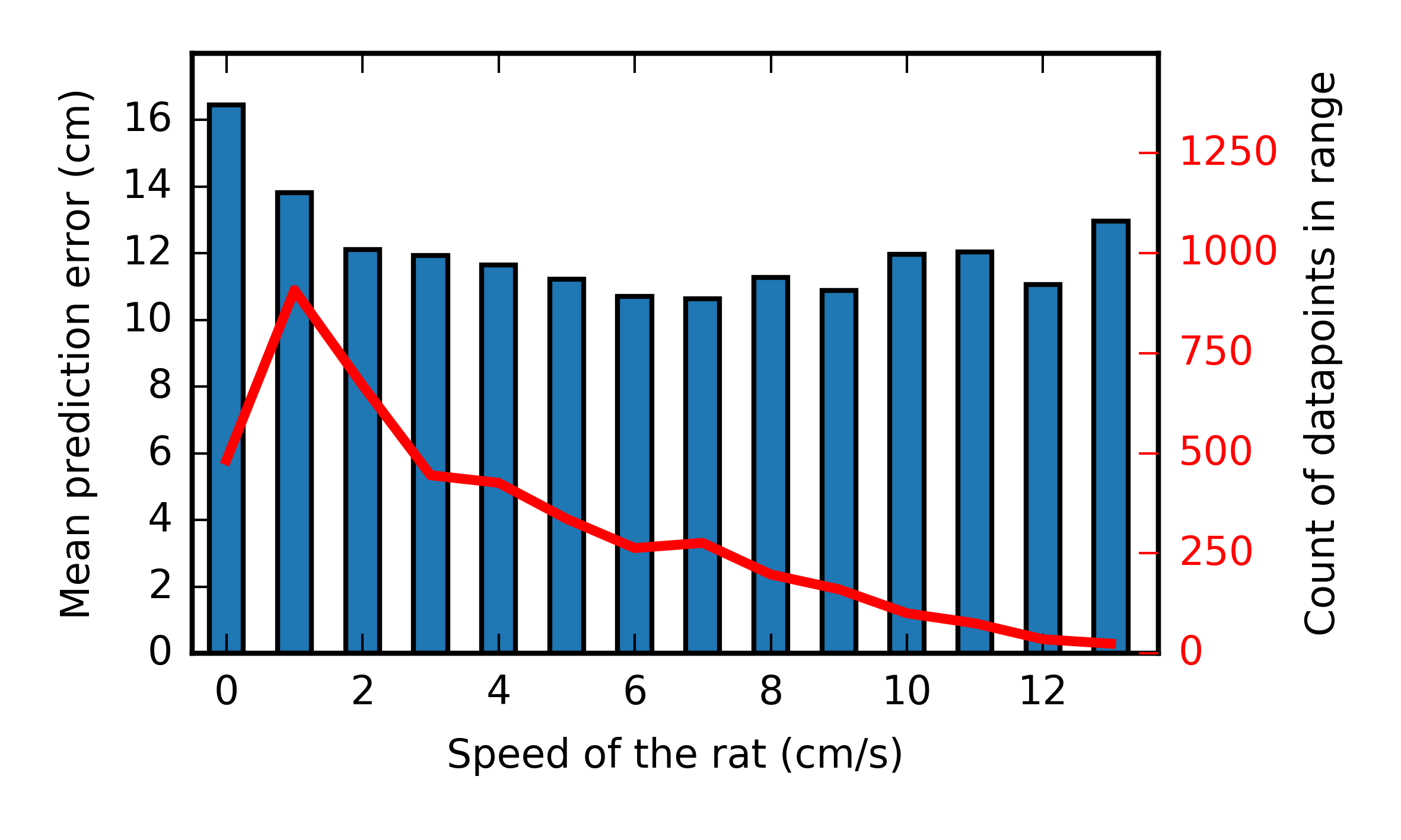

Supplement: S1 Fig — Movement speed is based on the distance covered in 200 ms. The first bar is the average over the errors for speeds in range [0, 0.5] cm/s, the second for (0.5, 1.5] cm/s, etc. The error is highest when the rat is not moving or moving very slowly. Notice that speeds in the range of 1-2 cm/s can also be the results of head movements. At higher speeds the exact velocity does not seem to influence accuracy. Note that the bars do not contain the same amount of data points. Apparent changes in the mean error at higher velocities can be attributed to noise as we have less data points there. (TIF) [file pcbi.1006822.s001.tif]

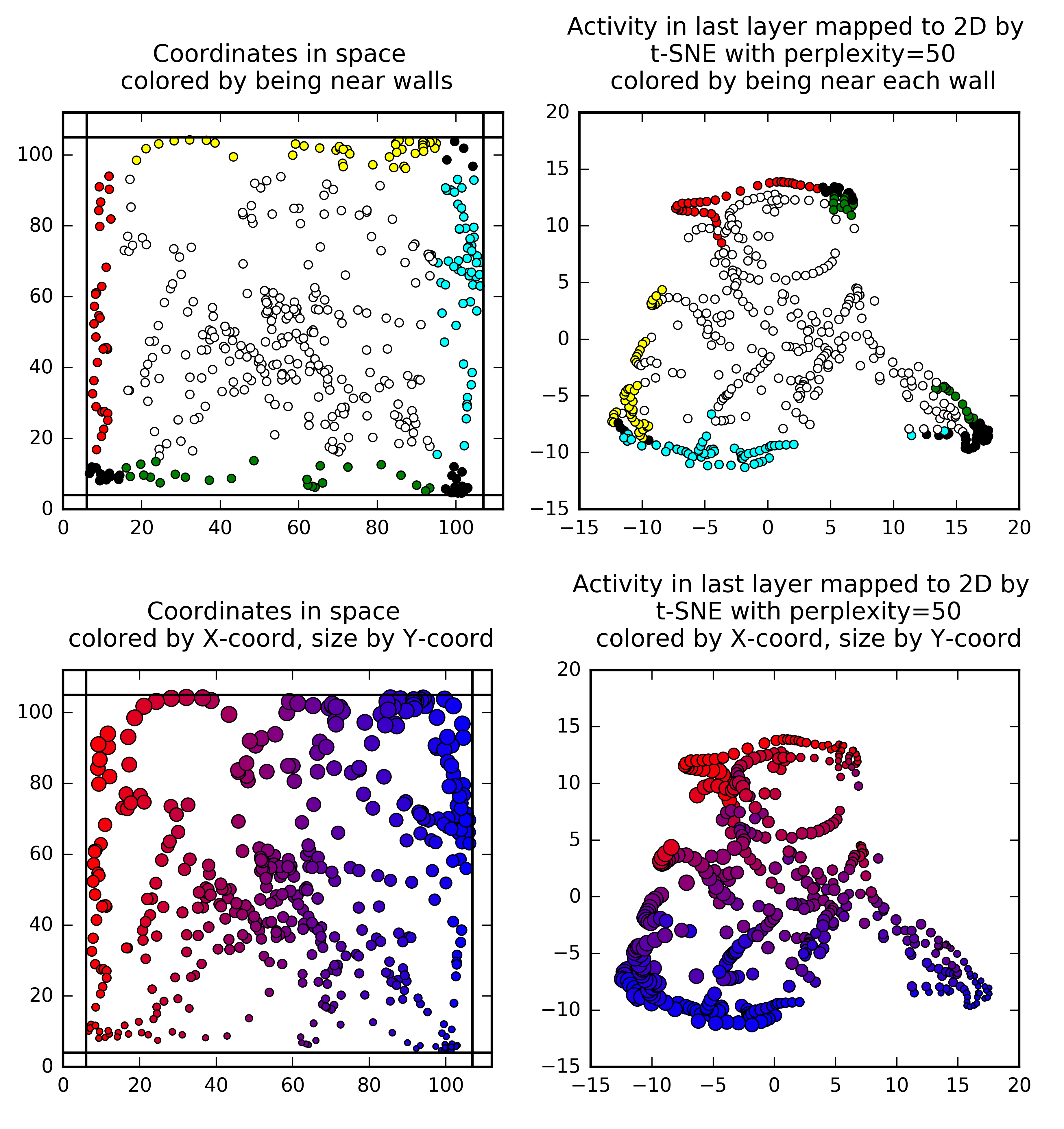

Supplement: S2 Fig — Top row: colouring reflects if the data point’s true location is near a wall. Bottom row: points are colored by the X-coordinate of true location of the data point and sized accoring to Y-coordinate. The left column illustrates how the the coloring schemes look on the true XY-coordinates. The right column shows the schemes applied on activations of nodes in the second layer of the RNN model at t = 100, reduced to 2D by t-SNE. Notice that data points with similar true locations are also nearby in activity space. (TIF) [file pcbi.1006822.s002.tif]

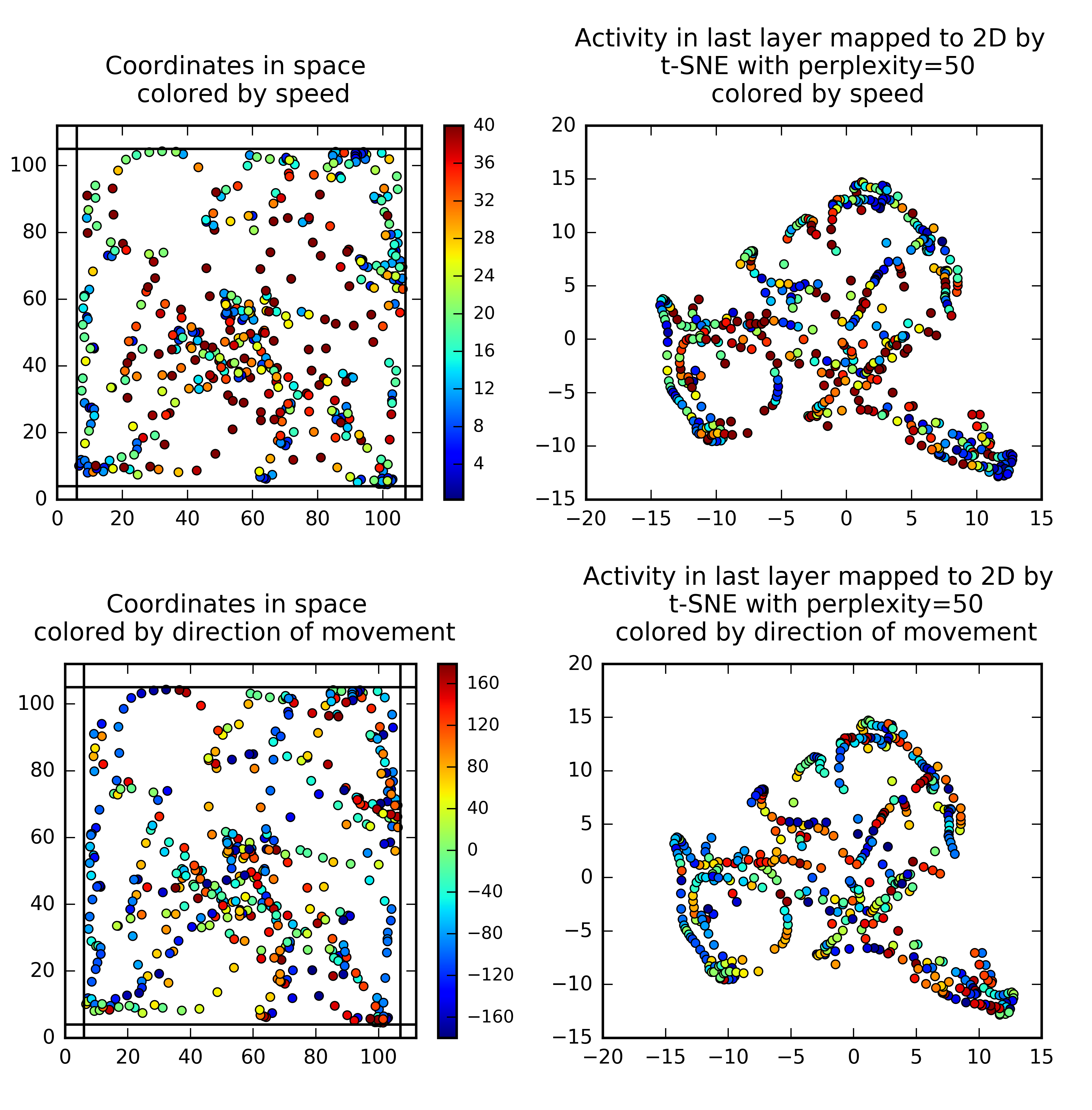

Supplement: S3 Fig — Top row: data points are colored according to instantaneous speed. Bottow row: data points a colored accoring to instantaneous direction of movement. Left column shows the correspondence between true location and speed and direction. In the right column the colouring schemes are applied to activations of nodes in the second layer of the RNN model at t = 100, reduced to 2D by t-SNE. There is no visible correspondence between RNN activations and neither speed nor direction. (TIF) [file pcbi.1006822.s003.tif]

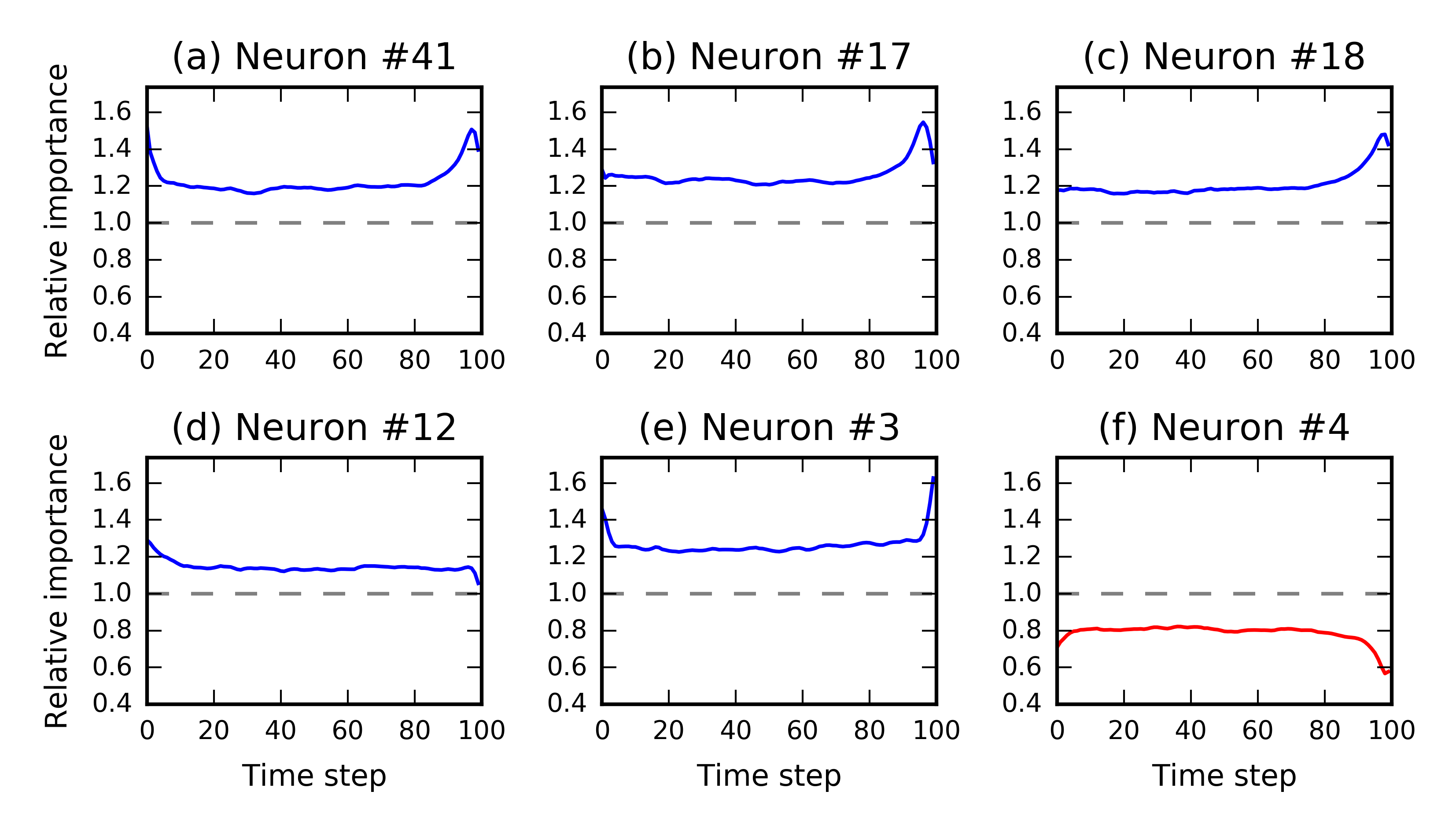

Supplement: S4 Fig — a-e) Temporal profiles of relative importance for 5 selected neurons among the highest contributing neurons according to gradient analysis. Notice that the profiles peak at different time steps. f) Temporal profile of the least important neuron according to gradient analysis. (TIF) [file pcbi.1006822.s004.tif]
