# Supplementary material for: Efficient neural decoding of self-location with a deep recurrent network
Source: PLoS Comput Biol. 2019 Feb 15;15(2):e1006822. doi: 10.1371/journal.pcbi.1006822 (PMC6407788; doi:10.1371/journal.pcbi.1006822)
Supplement: S1 Text — (PDF) [file pcbi.1006822.s005.pdf]

t-SNE analysis of RNN representations of input data

We visualized activations in the last recurrent layer of the RNN using the t-SNE dimensionality reduction technique (as implemented in Python's scikit-learn package). In particular, we extracted the node activations (i.e. how the input has been represented) of the second LSTM layer at the last timestep (t=100) for a number of data points. We then reduce these activity-vectors of 512 values to 2D using t-SNE. t-SNE iterative algorithm is run for 10000 steps and a wide range of perplexity values (5:400) are tested.

Because activity patterns are model specific it is not possible to compare data points originating from different neural network models. We therefore were restricted to using the 441 points comprising a single fold of the 10-fold cross validation. For the purpose of the visualizations on S2 Fig and S3 Fig, we used a fold (3rd fold) in which the animal visited all parts of the arena, including walls and corners.

First we investigated if the extracted activations reflect the animal's location in space. To this end we highlighted points corresponding to positions near to the environment walls. S2 Fig top left shows the points in XY-space (using true location) and top right shows the final layer activities of those same data points reduced to 2D by t-SNE. Data points corresponding to locations on the same wall also appear to be grouped together in activity space.

Second, we also visualized the correspondence directly between the t-SNE reduced activity-space and the true locations in 2D space using both color and marker size. Thus on the bottom row of S2 Fig, x-axis real-world location is indicated by color (red to blue) and y-axis by marker size (small to large), the bottom left plot demonstrates this color-size scheme projected onto 2D true location and the bottom right plot onto t-SNE reduced activity space. The fact that points of similar color and size are grouped together in t-SNE projections indicates that there is a correspondence between the locations in the activity space and true locations. This is also confirmed by Kendall's tau value between the distance matrices in the two spaces (t-SNE projection coordinates vs Euclidean coordinates of true locations; correlation 0.57, p-val<0.0001). It is perhaps not surprising that the last LSTM layer's activity space reflects the XY-coordinates because the output layer of the model linearly decodes animal position from these values.

Finally, we repeated the same visualization procedure colouring the points according to the animal's instantaneous movement speed and direction of travel (S3 Fig top and bottom row respectively). As before the left hand side of the figure shows these schemes projected on to the real world 2D coordinates and right hand side on to the t-SNE space. In neither case was there obvious correspondence between the color scheme and t-SNE activity.
