# Supplementary material for: Efficient neural decoding of self-location with a deep recurrent network
Source: PLoS Comput Biol. 2019 Feb 15;15(2):e1006822. doi: 10.1371/journal.pcbi.1006822 (PMC6407788; doi:10.1371/journal.pcbi.1006822)
Supplement: S2 Text — (PDF) [file pcbi.1006822.s006.pdf]

Temporal gradient analysis

A third way to investigate the gradients is to average only across the samples. We thus obtain an averaged gradient for each neuron at each different position in the input sequence of 100 time windows. These averages reveal, for example, how sensitive the model is to changes in spike counts of the same neuron at different points of time. Unfortunately recurrent network architecture and training procedures favour information contained in more recent inputs (due to vanishing gradients further back in time). We therefore judge that it is not fair to draw conclusions from comparing sensitivity to spike counts at different positions in the sequence - inputs in the later time steps would show up as more important not necessarily due to information content but due to the algorithm we used. It is however fair to compare the contributions of different neurons at the same time step. We propose to compare the model's sensitivity to a certain spike count with average of sensitivity across all neurons at the same point of the temporal context sequence. Intuitively such gradient analysis reveals if neuron N's activity at time window T within the temporal context, was more informative than the activity of other neurons at that time point. This comparison is not distorted by the network architecture, because inputs from different neurons are treated symmetrically (order of neurons could be changed) by the network. No bias exists with respect to either particular neurons or data samples.

As a summary of the analysis described above, S4 Figure shows the normalized gradients of several neurons at different positions within the temporal context. The analysis reveals different profiles of relative sensitivity within the temporal context. In particular, we note that several neurons have a peak in their normalized sensitivity around one second before the last time window for which the animal position is predicted. Nevertheless, our time windows last 1400ms and therefore the temporal resolution is very low. We restrain ourselves from drawing conclusions from this analysis due to lack of temporal precision. We believe that when using smaller, non-overlapping time windows, this type of investigation can reveal interesting temporal aspects of information processing in the brain.
